# Insignificant effect of Arctic amplification on the amplitude of midlatitude atmospheric waves

**DOI:** 10.1126/sciadv.aay2880

**Published:** 2020-02-19

**Authors:** Russell Blackport, James A. Screen

**Affiliations:** College of Engineering, Mathematics and Physical Sciences, University of Exeter, Exeter, UK.

## Abstract

Whether Arctic amplification has contributed to a wavier circulation and more frequent extreme weather in midlatitudes remains an open question. For two to three decades starting from the mid-1980s, accelerated Arctic warming and a reduced meridional near-surface temperature gradient coincided with a wavier circulation. However, waviness remains largely unchanged in model simulations featuring strong Arctic amplification. Here, we show that the previously reported trend toward a wavier circulation during autumn and winter has reversed in recent years, despite continued Arctic amplification, resulting in negligible multidecadal trends. Models capture the observed correspondence between a reduced temperature gradient and increased waviness on interannual to decadal time scales. However, model experiments in which a reduced temperature gradient is imposed do not feature increased wave amplitude. Our results strongly suggest that the observed and simulated covariability between waviness and temperature gradients on interannual to decadal time scales does not represent a forced response to Arctic amplification.

## Teaser

We find no evidence from observations or climate models that Arctic amplification causes a wavier midlatitude circulation.

## INTRODUCTION

Rising global temperatures are expected to increase the severity of certain types of extreme events such as heatwaves, droughts, and floods (*1*–*3*), primarily for well-established thermodynamical reasons. However, potential changes in weather extremes related to atmosphere dynamics, particularly over the midlatitudes, are far less certain (*4*, *5*). It has been proposed that the faster warming of the Arctic compared to the rest of world—so-called Arctic amplification—is altering the atmospheric circulation and contributing to an increase in extreme weather in the midlatitudes (*6*). One hypothesis proposed by Francis and Vavrus suggests that the reduced equator-to-pole temperature gradient weakens the predominant westerly wind, which, in turn, causes larger-amplitude waves in the midlatitude circulation (*7*, *8*), hereafter referred to as a “wavier” circulation. A wavier circulation has been linked to increased occurrence of extreme midlatitude weather, with the types of extremes favored by amplified waves varying by location (*9*). The link between Arctic amplification and a wavier midlatitude circulation remains controversial because of numerous studies arriving at often conflicting conclusions (*10*–*13*).

The purported evidence used to support the link between Arctic amplification and a wavier circulation stems primarily from observational analyses. The acceleration of Arctic warming for two to three decades starting from the mid-1980s coincided with a trend toward a wavier midlatitude circulation, particularly in autumn and winter (*7*, *8*). Furthermore, longitudes where there was a strong decrease in the meridional temperature gradient coincided with longitudes with increasing waviness (*8*). However, the metrics used to measure waviness have been questioned (*14*, *15*), and alternative metrics show that statistically robust trends are limited to few regions and seasons (*14*–*18*) and often only when more recent, short-term, trends are considered (*18*–*20*). The absence of statistically robust signals could be because Arctic amplification has only recently become of sufficiently large magnitude to have a detectable effect on the midlatitude circulation (*8*, *12*); thus, the effect is difficult to detect amidst the large internal atmospheric variability (*21*). Regardless of their statistical significance or not, the coincidence of observed trends in waviness and Arctic amplification may not mean that the relationship is causal.

Evidence for a causal response will likely have to come from theoretical arguments and modeling experiments. Basic theoretical arguments do not provide any unambiguous support for an increase in wave amplitudes under reduced meridional temperature gradients and zonal wind speeds (*4*, *22*). A decrease in wave amplitude was found in response to Arctic amplification in experiments with a highly idealized model but which retained the essential physics required in the hypothesis proposed by Francis and Vavrus (*23*). This decrease in wave amplitude occurred because of weaker synoptic variability (*22*, *24*) in midlatitudes and despite a mean reduction in the zonal wind speed. Numerous studies have used more complex climate models to test whether we might expect to see a wavier circulation in the future. In contrast to the Francis and Vavrus hypothesis, models forced with increasing greenhouse gas concentrations show a small decrease in waviness, albeit with substantial intermodel spread (*18*, *25*, *26*). In addition to the effect of reduced variability, this waviness decrease may be partly attributed to an increase in the meridional temperature gradient aloft, which tends to oppose the midlatitude circulation response to a decreased meridional temperature gradient near the Earth’s surface (*10*, *18*, *25*). Model experiments forced with Arctic amplification in isolation find only a weak response in waviness compared to internal variability (*27*–*29*). Other aspects of the large-scale circulation also show only weak responses, compared to internal variability, in model experiments forced with observed sea ice loss (*30*–*32*). Overall, model simulations do not support a causal link between Arctic amplification and increased waviness and, instead, suggest that the observed increase in waviness was a result of internal variability and is unlikely to continue. It is possible that the models are wrong because of deficiencies in simulating the relevant processes, but direct evaluation of the models’ capability in reproducing the observed links between Arctic amplification and waviness has not been undertaken.

Despite substantial scientific uncertainty, the Francis and Vavrus hypothesis has become a regular narrative in media reporting of extreme weather events (*33*–*35*). This widespread media reporting is likely a major reason why there is high public belief that if Arctic warming continues, it will have major effects on midlatitude weather (*33*). Some scientists argue that the possible effects of Arctic amplification on the circulation have been overstated in the public discourse and distracted from other more certain and no less concerning consequences of climate change (*36*).

Previous work examining changes in waviness in response to Arctic amplification has focused on either only observations (*7*, *8*, *19*, *20*) or only models (*23*, *25*–*29*) or compared recently observed trends to future model projections (*18*), making fair model-observation comparisons difficult. Here, we attempt to reconcile the divergent conclusions of previous studies by making “like-for-like” comparisons between observations and models. First, we update the observed waviness trends to the end of 2018 to examine whether the previously reported increases (*7*, *8*, *18*, *19*) have continued and compare them to the range of simulated trends from a multimodel large ensemble. Next, we examine the correspondence between Arctic amplification and waviness as manifested in interannual to decadal variability in both observations and models. Last, we perform controlled model experiments to determine the direction of causality in simulated relationships between Arctic amplification and waviness.

## RESULTS

### Recently observed trends

**Fig. 1:**
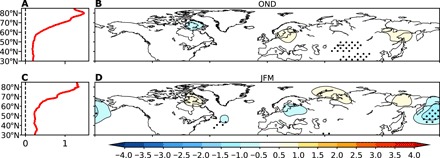
Observed trends in near-surface temperature and waviness. (**A**) Trends in zonal mean SAT (°C per decade) as a function of latitude during OND from 1979 to 2018 in ERA-Interim reanalysis. (**B**) Observed trends in LWA (10^7^ m^2^ per decade) during OND from 1979 to 2018. Stippling indicates trends that are significant at the 95% confidence interval level. (**C** and **D**) As in (A) and (B), but for JFM.

We begin by investigating the observed trends in near-surface air temperature (SAT) and the waviness of the midlatitude circulation from the ERA-Interim reanalysis over the 1979–2018 period. Figure 1 (A and C) shows the trends in zonal mean SAT in October-November-December (OND) and January-February-March (JFM). During both seasons, the amplified Arctic warming is clear, with Arctic temperatures (north of 65°N) rising about four times faster than in midlatitudes. To quantify the waviness, we use the local wave activity (LWA) (*19*, *20*, *37*), which measures the meridional extent and magnitude of displacements in daily averaged 500-hPa geopotential height contours (see Materials and Methods). Large LWA is closely linked to regional weather extremes, such as warm and cold temperature extremes (*19*), and is associated with blocking (*20*). Unlike many previous metrics used to measure waviness, the LWA can be calculated at each latitude and longitude, allowing us to examine regional trends without having to make arbitrary regional definitions. Despite the clear Arctic amplification and reduced meridional temperature gradient over the past 40 years, there is little change in LWA at either hemispheric or regional scales (Fig. 1, B and D). There is not a single grid point over the midlatitudes in either OND or JFM, where a statistically significant increase in LWA is found. The largest magnitude trends are reductions over the North Pacific during JFM. Plots of the zonal mean LWA anomaly as a function of year and latitude show large variability on interannual and decadal time scales, but no clear increase over the past 40 years (fig. S1), in agreement with the linear trends. Thus, we find no evidence of a wavier midlatitude circulation in response to Arctic amplification in the observed trends from 1979 to 2018.

**Fig. 2:**
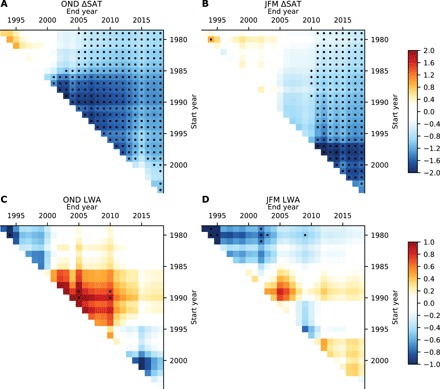
Observed trends in the meridional near-surface temperature gradient and waviness as a function of start and end year. (**A**) Trends in ΔSAT (°C per decade) during OND as a function of start year (vertical axis) and end year (horizontal axis). Only trends of greater than 15 years in length are plotted. Stippling indicates trends that are statistically significant at the 95% confidence interval level. (**B**) As in (A), but for JFM. (**C**) As in (A), but for the LWA (SDs per decade) averaged over 40° to 60°N. (**D**) As in (C), but for JFM.

Some previous studies have found increases in waviness over shorter time periods (*7*, *8*, *18*, *19*, *38*), so we next examine the effect of changing the start and end year when calculating trends. We use the difference between midlatitude (30° to 50°N) and Arctic (north of 65°N) SAT (ΔSAT) as a measure of the meridional temperature gradient and of Arctic amplification. Note that a decrease in ΔSAT corresponds to reduced meridional temperature gradient and thus to Arctic amplification. Figure 2A shows the linear trend in ΔSAT as a function of start year and end year for OND over the 1979–2018 period. Trends in ΔSAT starting after 1979 became statistically significant around 2005 and have continued to increase in magnitude and statistical significance since. Varying the start year has little effect on the statistical significance of the trends, as all trends starting before 1993 and ending in 2018 are highly statistically significant (*P* < 10^−6^). Similar results are found for JFM (Fig. 1B), but the trends in ΔSAT became statistically significant more recently (around 2010). Several past studies have chosen, for often unspecified reasons, to examine trends starting around 1990 when investigating trends in waviness or other aspects of Arctic-to-midlatitude linkages (*6*, *19*, *20*, *38*, *39*). One study claims that Arctic amplification emerged around 1990 (*8*); however, we find that statistically robust trends in ΔSAT are found when starting trends before 1990, and there is nothing special about 1990. Thus, we argue that there is no justification for neglecting the earlier years based on ΔSAT.

Trends as a function of start and end year for LWA averaged over all longitudes and 40° to 60°N are plotted in Fig. 2 (C and D) for OND and JFM, respectively. During OND, we find an increase in LWA from about 1990 to 2010, consistent with previous work (*7*). However, this trend disappears when updating trends with the most recent data or going back further to 1979, resulting in very weak and not statistically significant multidecadal trends in LWA (0.02 ± 0.28 SDs per decade over 1979–2018). In contrast to the Francis and Vavrus hypothesis, the absence of a multidecadal trend in LWA occurs despite the ΔSAT trend being very highly statistically significant (*P* < 10^−7^) over the entire period. There were also increases in LWA in JFM for trends starting around 1990 (Fig. 1D), albeit weaker in magnitude than in OND and not statistically significant, but these also disappear when looking at the full period. From 1979 to 2018, there is a small, but not statistically significant, decrease in LWA (−0.17 ± 0.28 SDs per decade). During JFM, the positive trends and subsequent reversal are more apparent when examined only over the North America–Atlantic region (fig. S2), which was the region of focus in one prominent study (*7*). Larger magnitude and statistically significant regional increases are found when examining shorter-term trends (fig. S3), consistent with previous studies (*19*, *20*, *38*). However, similar magnitude regional decreases can be found over different time periods of similar length (fig. S3). As these regional trends are not seen in longer-term trends (Fig. 1), they likely reflect internal variability.

One potential source of discrepancy between studies is that different metrics can give different answers (*15*, *28*), so we have calculated trends in waviness using two additional metrics: the meridional circulation index (*8*) and sinuosity (*18*). All three metrics are in agreement that there has been no significant change in waviness over the past 40 years (fig. S4). The observed trends in April-May-June (AMJ) and July-August-September (JAS) using all three metrics are also shown in fig. S4. While the focus of our study is on autumn and winter, it has also been proposed that Arctic amplification could increase wave amplitudes during summer through different mechanisms than discussed here (*40*). All metrics show small reductions in waviness during both AMJ and JAS, although the changes are not statistically significant in either season (fig. S4).

### Model-observation comparisons

**Fig. 3:**
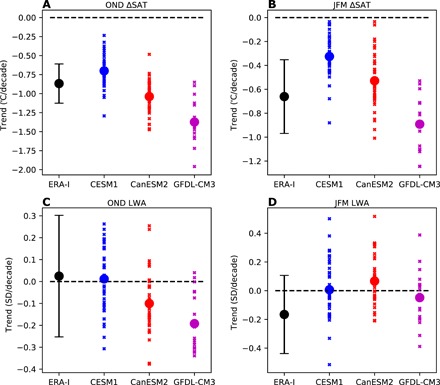
Comparison of observed and simulated trends in the meridional near-surface temperature gradient and waviness. (**A**) Trend in ΔSAT (°C per decade) during OND from 1979 to 2018 in ERA-Interim reanalysis (black) and the three models (blue, red, and magenta). Error bars for ERA-Interim indicate the 95% confidence interval. For the models, the small crosses indicate the trends in individual ensemble members, and large dots indicate the trend of the ensemble mean. (**B**) As in (A), but for JFM. (**C**) As in (A), but for LWA (SDs per decade). (**D**) As in (C), but for JFM.

We now compare the observed 1979–2018 trends in ΔSAT and LWA to simulated trends over the same time period from large initial condition ensembles using three independent climate models (see Materials and Methods). As the forcing is identical in each ensemble member, the ensemble spread in each model represents the range of possible outcomes only due to internal variability. There is good agreement between the observed and modeled multidecadal ΔSAT trends: The spread within each ensemble overlaps with the observed trends during OND and JFM (Fig. 3, A and B, respectively). The ensemble means from all three models show statistically significant downward trends in ΔSAT. For LWA, the spread within each ensemble also overlaps with the observed multidecadal trends during OND and JFM (Fig. 3, C and D). In all cases, the spread of the modeled LWA trends crosses zero, consistent with the statistically insignificant observed multidecadal trends. Recall that we found a statistically significant increase in observed LWA for the 21-year period 1990–2010 during OND. We find that all models have at least one ensemble member with a 21-year trend as large as the observed increase from 1990 to 2010 during OND. Thus, we find no evidence of a discrepancy between observed and modeled ΔSAT or LWA trends. The largest magnitude simulated LWA trends are found during OND in the GFDL-CM3 model, which shows a decrease in LWA over the full 40-year period. This reduction in LWA occurs despite this model also having the largest reductions in ΔSAT, opposite to what would be predicted on the basis of the Francis and Vavrus hypothesis.

**Fig. 4:**
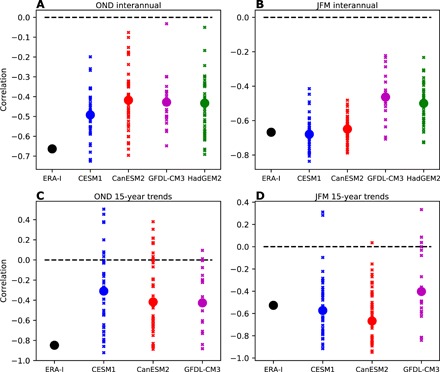
Correlations between the meridional near-surface temperature gradient and waviness in internal variability. (**A**) Correlations between ΔSAT and LWA in interannual variability during OND for ERA-Interim reanalysis (black) and the four models (blue, red, magenta, and green). For the models, the small crosses indicate the correlation in individual ensemble members, and the large dots indicate the correlation for concatenated time series of all ensemble members. (**B**) As in (A), but for JFM. (**C**) As in (A), but for correlations of 15-year overlapping trends. (**D**) As in (C), but for JFM.

Although Figs. 1 to 3 show no clear correspondence between ΔSAT and LWA on multidecadal time scales, trends in the two quantities appear to vary coherently on decadal and shorter time scales (Fig. 2), motivating closer examination. Correlations between ΔSAT and LWA on interannual time scales in observations and in the models are shown in Fig. 4 (A and B) for OND and JFM, respectively. Here, we have also included data from an additional model (HadGEM2) that will be used later for controlled experiments. More specifically, here, we include in our analysis a set of short, 5-year-long simulations with HadGEM2 (see Materials and Methods), which cannot be used to examine trends but is useful for examining interannual variability. In OND, there is a strong correlation between the ΔSAT and LWA in observations (−0.66) and weaker, but still statistically significant, correlations in each of the four models (−0.49, −0.42, −0.43, and −0.43). In three of the four models, the observed correlation is within the ensemble spread, albeit on the high end. Similar results are found for correlations between overlapping 15-year trends (Fig. 4C), with the observed values again being on the high end of the model spread. During JFM (Fig. 4, B and D), the observed correlations are closer to the middle of the ensemble range from the models, with an observed interannual correlation of −0.67 and statistically significant correlations of −0.68, −0.65, −0.46, and −0.50 in each of the four models. This implies that time periods with smaller decadal decreases in ΔSAT are more likely to have negative decadal trends in LWA. Observed correlations are within the ensemble range for all models for both interannual variability and 15-year trends during JFM.

**Fig. 5:**
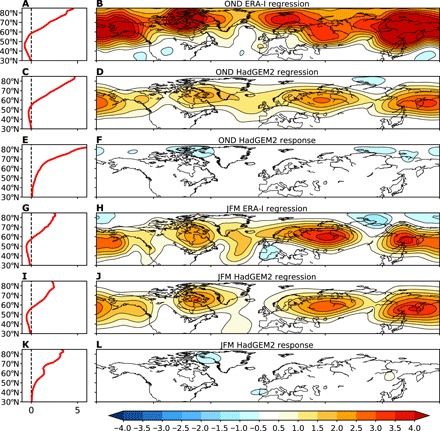
Links between the meridional near-surface temperature gradient and waviness in internal variability versus the forced response. (**A**) Zonal mean SAT (°C) regressed onto ΔSAT during OND for ERA-Interim. (**B**) LWA (10^7^ m^2^) regressed onto ΔSAT during OND for ERA-Interim. (**C** and **D**) As in (A) and (B), but for the HadGEM2 model. (**E**) The zonal mean near-surface temperature (°C) response to Arctic amplification in HadGEM2. (**F**) Response of LWA (10^7^ m^2^) to Arctic amplification in the HadGEM2 simulations. (**G** to **L**) As in (A) to (F), but for JFM. The magnitudes of the regression coefficients are scaled by the ΔSAT response in the experiments forced with Arctic amplification (3.06° and 2.17°C in OND and JFM, respectively).

Next, we examine the regional structure of the LWA associated with a reduction in ΔSAT in observations (Fig. 5, B and H) and in HadGEM2 (Fig. 5, D and J) by regressing the LWA onto the ΔSAT in interannual variability (with the sign reversed). In both ERA-Interim and HadGEM2, a decrease in ΔSAT is associated with increased LWA throughout the midlatitudes, with peaks occurring over the North Pacific, northeastern Canada, and the Ural mountain region. In OND (Fig. 5, B and D), the magnitude is stronger in observations, particularly over the high latitudes, while there is closer agreement in JFM (Fig. 5, H and J), consistent with the correlations found in Fig. 4. Thus, the model captures the observed associations between the spatial structure of LWA and ΔSAT on interannual time scales. The regressions of the corresponding zonal mean SAT onto the ΔSAT are shown in Fig. 5 (A, C, G, and I). By construction, a decrease in ΔSAT is associated with strong warming in the Arctic and weak cooling over the midlatitudes.

### Links in internal variability versus the forced response

The relationship between the LWA and ΔSAT on interannual to decadal time scales in both models and observations might suggest a causal relationship. However, internal variability cannot always be used to predict the forced response (*41*), and causality could operate in either direction: Changes in ΔSAT could cause changes in waviness, or changes in waviness could cause changes in ΔSAT. To test the causality of the relationship, we forced the HadGEM2 model with Arctic amplification (Fig. 5, E and K) by reducing the sea ice (see Materials and Methods) and examined the response in LWA (Fig. 5, F and L). While sea ice loss by itself in atmosphere-only simulations may not capture the vertical structure of the Arctic temperature response to global warming (*11*), it does in the coupled atmosphere-ocean simulations used here (fig. S5), in agreement with other coupled model experiments (*29*, *42*, *43*). Despite the strong associations between LWA and ΔSAT in the model (Fig. 5, D and J), when ΔSAT is forced, there is little LWA response over any region in either OND or JFM. Note that the regressions have been scaled by the ΔSAT forced in the sea ice loss simulations (3.06° and 2.17°C in OND and JFM, respectively) so that magnitudes between the regressions and the response can be easily compared. In addition, note that the ΔSAT forced in the simulations is similar in magnitude to what has been seen in the recent observations (3.47°C/40 years and 2.64°C/40 years in OND and JFM, respectively). Thus, while the model shows a clear connection between ΔSAT and LWA on interannual time scales, changes in LWA do not appear to be caused by changes in ΔSAT. This is consistent with observations that also show clear connections on interannual to decadal time scales but no correspondence over the 40-year observed record.

If the correspondence between LWA and ΔSAT seen on interannual to decadal time scales is not being forced by ΔSAT, then what is causing the relationship? The LWA is an associated anomalous meridional eddy heat flux (*19*); therefore, an increase in LWA will force an increase in high-latitude temperatures and a decrease in midlatitude temperatures. Furthermore, extreme moisture transport into the Arctic, which can cause strong warming, is associated with wavy midlatitude circulation features such as blocking (*44*) and Rossby wave breaking (*45*). We argue that it is likely that the causality goes in the opposite direction: Internal variability in the LWA causes anomalous changes in ΔSAT. This interpretation is supported by daily lead-lag correlations from the HadGEM2 model, which show that the strongest correlations occur when LWA leads ΔSAT (fig. S6). We cannot fully rule out the possibility that internal variability in ΔSAT on interannual to decadal time scales can contribute to variability in LWA on these time scales, despite the lack of a forced LWA response to ΔSAT on multidecadal time scales. However, the physical mechanisms that might explain such a distinction between time scales are unclear.

## DISCUSSION

Our results help to resolve the apparent discrepancy between the observed increased in waviness and the small decrease projected by modeling studies. In the years since the observed increase was first detected (*7*), Arctic amplification has continued; however, the increase in waviness has not. Over the past 40 years, seasonal trends in waviness across all regions and using multiple metrics are close to zero, in agreement with multidecadal trends simulated by models. This strongly suggests that the previously reported increases in waviness were a manifestation of internal variability. We have shown that in both observations and models, there is a correspondence between changes in the meridional temperature gradient and the waviness of the midlatitude circulation on interannual to decadal time scales. However, this correspondence is not seen over the 40-year observed and modeled trends or in model experiments forced with a reduced temperature gradient. We conclude that the association is not indicative of a forced response of waviness to Arctic amplification, and instead, it likely arises because of internal climate variability. We further speculate that the relationship between interannual to decadal changes in the meridional temperature gradient and the waviness of the midlatitude circulation is not simply a random occurrence of internal variability but instead partly occurs because the changes in waviness cause changes in the meridional temperature gradient, consistent with physical expectations (*19*, *44*, *45*). The combination of internal variability and misinterpretation of causality could potentially explain the discrepancy between observational and modeling studies.

Our results have important implications for interpreting the coincidence of increased waviness with accelerated Arctic warming. Several past studies have inferred a circulation response to Arctic amplification by examining trends starting from around 1990 (*6*, *19*, *38*, *39*), motivated by the acceleration in Arctic amplification at this time, to the mid-2010s, when the analyses were conducted. However, on these relatively short time scales, internal variability in the atmospheric circulation may have contributed to the more rapid Arctic amplification. Thus, the trends observed in the midlatitudes over these short periods may instead be associated with the circulation driving the more rapid Arctic warming and may not be a response to Arctic amplification. We therefore urge caution when interpreting the links between Arctic amplification and the midlatitude circulation based on short-term trends, and advocate for using the full period of observations, as this will provide a more robust estimate of the forced response. We reiterate that Arctic amplification is detectable in the observed record when starting trends well before 1990, and arguments that Arctic amplification has only emerged since 1990 appear misguided.

If Arctic amplification is not a cause of increased waviness, a logical next question to ask is where in the proposed chain of causality does the Francis and Vavrus hypothesis break down. Recall that the hypothesis states that Arctic amplification reduces the westerly wind and that a slower flow is wavier. In our simulations, we do find a modest but statistically significant decrease in strength of the westerly winds over mid- and high latitudes in response to Arctic amplification, consistent with similar modeling experiments using other models and protocols (*46*). So, the proposed connection between Arctic amplification and a slower westerly flow appears sound, at least qualitatively. However, a slower westerly flow forced by Arctic amplification does not result in a wavier circulation. This appears to be the weak link in the proposed chain. Changes in wave amplitude are governed by factors in addition to the westerly wind strength, including baroclinicity, moisture, lower tropospheric heating, and tropical wave driving (*4*).

In summary, we find no significant effect of Arctic amplification on the waviness of the midlatitude circulation in observations or models. The correspondence between Arctic amplification and waviness on interannual to decadal time scales is not indicative of a forced response of waviness to Arctic amplification and likely arises because internal variability in the midlatitude circulation causes changes in the meridional temperature gradient. Thus, future Arctic amplification is unlikely to cause a wavier midlatitude circulation or an increase in dynamically driven extreme weather. The impact of Arctic amplification on midlatitude temperature extremes during autumn and winter will likely be dominated by thermodynamic effects, which are very robust in models (*24*) and are grounded in well-established theory (*22*).

## MATERIALS AND METHODS

### Reanalysis data

For observations, we used the monthly averaged 2-m temperature and 6-hourly 500-hPa geopotential height, meridional wind, and zonal wind from the ERA-Interim reanalysis (*47*) for the period 1979–2018. The daily average of the 6-hourly 500-hPa geopotential heights was calculated before any calculations were done, to be consistent with the model analyses.

### Model experiments

We used data from initial condition large ensembles from three different models for the period 1979–2018. The three models are CESM1 (40 members) (*48*), CanESM2 (50 members) (*49*), and GFDL-CM3 (20 members) (*32*). These are coupled atmosphere-ocean models, which simulate interactions between the atmosphere, ocean, sea ice, and land surface. All ensemble members were branched off a historical forced simulation well before 1979 (1920, 1950, and 1920 for each model, respectively). Each member differs by only small changes in initial conditions; thus, any differences between members reflect only internal variability. The simulations were forced by historical forcing until 2005, followed by RCP8.5 (Representative Concentration Pathway 8.5) until 2018.

The HadGEM2 simulations are the same as those used by Blackport and Screen (*50*), where a more detailed description can be found. These include a present-day control ensemble that consists of 400 realizations that are 5 years in length that use RCP8.5 forcing from 2008 to 2012. The only differences between ensemble members were the initial conditions. The first year is discarded, so the full ensemble used for analysis consists of 1600 years. For comparison to the interannual correlations in observations and other model simulations in Fig. 4, correlations were calculated over 40 separate 40-year segments, in addition to the full 1600 years. To investigate the response to Arctic amplification, we ran an additional ensemble that was identical to the present-day control ensemble, but the sea ice was reduced by altering the sea ice albedo. Specifically, the albedo of cold deep snow was reduced from 0.80 to 0.05, and the albedo of snow-free ice was increased from 0.61 to 0.66. The small increase in snow-free sea ice resulted in a more realistic seasonal cycle of sea ice reduction. The response to Arctic amplification shown in Fig. 5 (E, F, K, and L) is the difference between the ensemble with the reduced sea ice and the present-day control ensemble. For the comparison of the temperature response in the simulations with reduced sea ice to the response to global warming in fig. S5, a third ensemble was used, which was identical to the present-day control ensemble, but the RCP8.5 forcing from 2036 to 2040 was used. The changes in ΔSAT in the ensembles with reduced sea ice and global warming were nearly identical (3.06° and 3.00°C in OND and 2.17° and 2.14°C in JFM).

### Waviness metrics

Most of our analysis of the waviness of the midlatitude circulation used the LWA, which was first introduced by Huang and Nakamura (*37*) using potential vorticity and has since been used with the 500-hPa geopotential height (*19*, *20*), as was done here. We followed the methods of Chen *et al.* (*19*) and Martineau *et al.* (*20*), where more details can be found. First, we calculated the equivalent latitude (ϕ_e_) for a given line of constant geopotential height, *z*_c_

$$\phi_{e}(z_{c})={\text{sin}}^{-1}(1-\frac{\iint_{z\leq z_{c}}\text{cos}\;\phi \;d\lambda d\phi }{2\pi })$$ (1)

where *z* is the geopotential height, ϕ is the latitude, and λ is the longitude. This represents the latitude for which the total area enclosed between the latitude and the pole is equivalent to the area where *z* is smaller than *z*_c_. The equivalent latitudes were calculated separately each day to avoid artifacts associated with shifts in heights with seasons or global warming. We then calculated the anticyclonic LWA (LWA_A_) and cyclonic LWA (LWA_C_) for each latitude and longitude

$${\text{LWA}}_{A}(\lambda ,\phi_{e})=\frac{a}{\text{cos}\;\phi_{e}}\int_{\hat{z}\geq 0,\phi \geq \phi_{e}(z_{c})}\hat{z}(\lambda ,\phi )\text{cos}\;\phi \;d\phi$$ (2)

$${\text{LWA}}_{C}(\lambda ,\phi_{e})=-\frac{a}{\text{cos}\;\phi_{e}}\int_{\hat{z}\leq 0,\phi \leq \phi_{e}(z_{c})}\hat{z}(\lambda ,\phi )\text{cos}\;\phi \;d\phi$$ (3)

where $\hat{z}=z-z_{C}$ and *a* is the radius of Earth. The anticyclonic and cyclonic components were then summed to get the total LWA. Thus, the LWA at each grid point measures the extent and magnitude of excursions of geopotential height anomalies to the north (ridges) and south (troughs), including any cutoff highs and lows. The LWA was calculated for each day from daily averaged 500-hPa geopotential height data. For the trends in Figs. 2 and 3, the LWA was normalized by the observed SD.

In fig. S4, we also computed the meridional circulation index (MCI) introduced by Francis and Vavrus (*8*), which measures the ratio of the meridional wind speeds to the total wind speed

$$\text{MCI}=|\frac{v*|v|}{u^{2}+v^{2}}|$$ (4)

where *u* and *v* are the zonal and meridional components of the wind speed, respectively. This was calculated at each grid point from daily *u* and *v* data and then averaged over the season, all longitudes and from latitudes 40° to 60°N.

Last, we also used the sinuosity metric introduced by Cattiaux *et al.* (*18*). For each day, the average 500-hPa geopotential height between 30° and 70°N was calculated to find the line of constant geopotential height that corresponds to approximately 50°N. The sinuosity was calculated as the total perimeter of this line of constant geopotential height, including any cutoff highs and lows. The daily values were then averaged over each season.

### Correlation analysis

All the reanalysis data were linearly detrended before calculating the correlations and regressions in Figs. 4 and 5. To be consistent with the observational analysis, we removed the linear trend in each ensemble member before calculating the correlations. Similar results were found if instead the ensemble mean was removed. Similar results were also obtained if the data were not detrended before calculating the correlations, but correlations and regression coefficients were slightly weaker. For the correlations of the 15-year trends in Fig. 4 (C and D), we calculated the trends for each overlapping 15-year period in the observations and models. This results in 26 different trend values in the 40-year observed record and each ensemble member from the simulations. The correlation between these 26 different LWA and ΔSAT values was then calculated.

## Supplementary Material

http://advances.sciencemag.org/cgi/content/full/6/8/eaay2880/DC1

Download PDF

Insignificant effect of Arctic amplification on the amplitude of midlatitude atmospheric waves

## SUPPLEMENTARY MATERIALS

Supplementary material for this article is available at http://advances.sciencemag.org/cgi/content/full/6/8/eaay2880/DC1 Fig. S1. Observed waviness as a function of latitude and year. Fig. S2. Observed trends in waviness over the North American–Atlantic region. Fig. S3. Short-term observed trends in waviness. Fig. S4. Observed trends in waviness from additional metrics across all seasons. Fig. S5. Zonal mean temperature response to sea ice loss and global warming. Fig. S6. Daily lead-lag correlations between waviness and meridional near-surface temperature gradient.
