# Supplementary material for: Insignificant effect of Arctic amplification on the amplitude of midlatitude atmospheric waves
Source: Sci Adv. 2020 Feb 19;6(8):eaay2880. doi: 10.1126/sciadv.aay2880 (PMC7030927; doi:10.1126/sciadv.aay2880)
Supplement: http://advances.sciencemag.org/cgi/content/full/6/8/eaay2880/DC1 [file supp_6_8_eaay2880__index.html]

Science Advances | Science AdvancesAAASSearchScience AdvancesMenu

## Supplementary Materials

**This PDF file includes:**

- Fig. S1. Observed waviness as a function of latitude and year.
- Fig. S2. Observed trends in waviness over the North American–Atlantic region.
- Fig. S3. Short-term observed trends in waviness.
- Fig. S4. Observed trends in waviness from additional metrics across all seasons.
- Fig. S5. Zonal mean temperature response to sea ice loss and global warming.
- Fig. S6. Daily lead-lag correlations between waviness and meridional near-surface temperature gradient.

Download PDF

**Files in this Data Supplement:**

- Adobe PDF - aay2880\_SM.pdf
