# Supplementary material for: Insignificant effect of Arctic amplification on the amplitude of midlatitude atmospheric waves
Source: Sci Adv. 2020 Feb 19;6(8):eaay2880. doi: 10.1126/sciadv.aay2880 (PMC7030927; doi:10.1126/sciadv.aay2880)
Supplement: Download PDF [file aay2880_SM.pdf]

Science Advances
AAAS

advances.sciencemag.org/cgi/content/full/6/8/eaay2880/DC1

Supplementary Materials for

Insignificant effect of Arctic amplification on the amplitude of midlatitude atmospheric waves

Russell Blackport* and James A. Screen

*Corresponding author. Email: r.blackport@exeter.ac.uk

Published 19 February 2020, *Sci. Adv.* **6**, eaay2880 (2020)
DOI: 10.1126/sciadv.aay2880

This PDF file includes:

Fig. S1. Observed waviness as a function of latitude and year.
Fig. S2. Observed trends in waviness over the North American–Atlantic region.
Fig. S3. Short-term observed trends in waviness.
Fig. S4. Observed trends in waviness from additional metrics across all seasons.
Fig. S5. Zonal mean temperature response to sea ice loss and global warming.
Fig. S6. Daily lead-lag correlations between waviness and meridional near-surface temperature gradient.

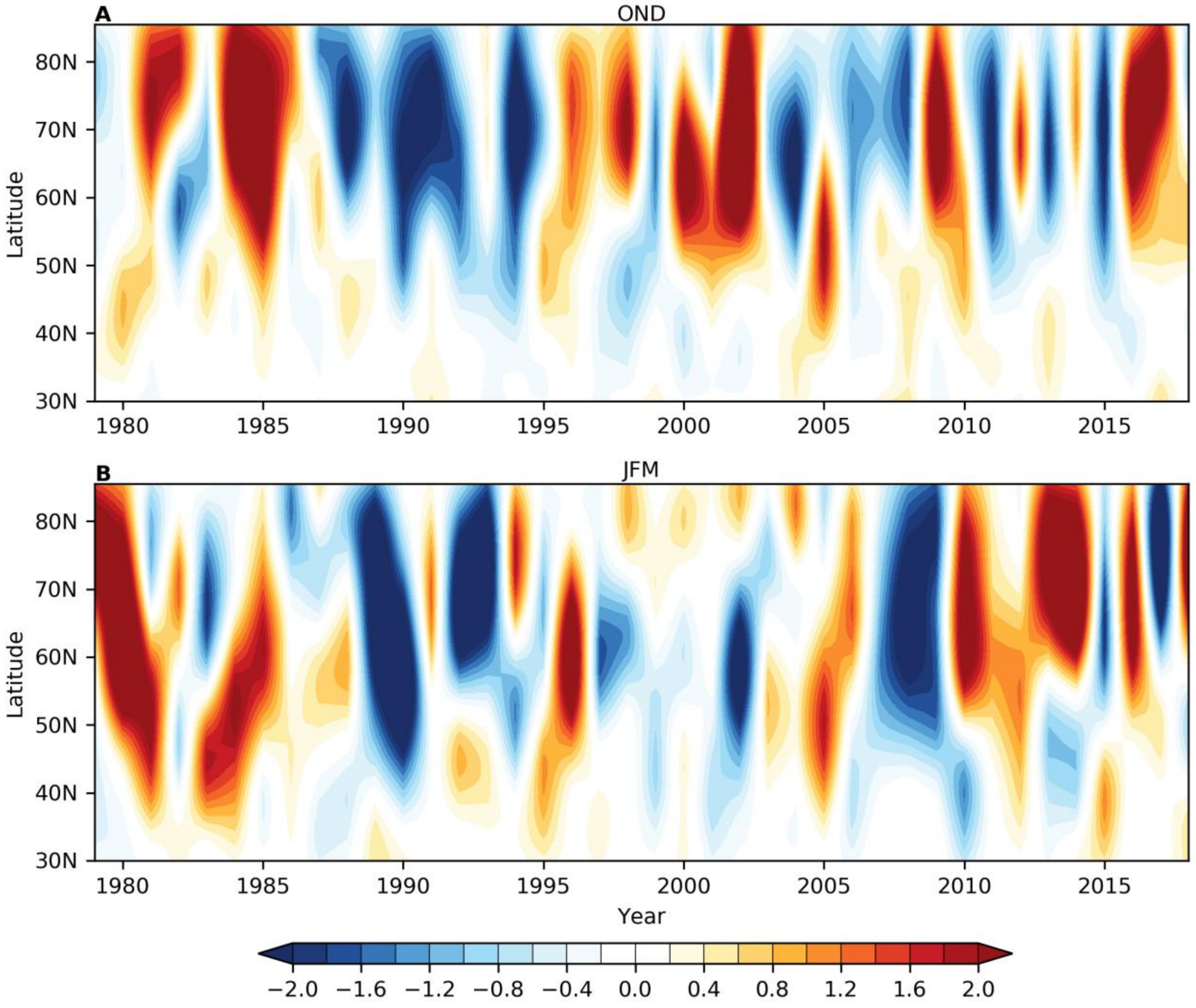

Fig. S1. Observed waviness as a function of latitude and year. (A) Zonal mean LWA anomaly (10^7 m^2) as function of latitude and year during OND. Anomalies were calculated by subtracting the 1979-2018 mean at each latitude separately. (**B**) As in (A), but for JFM.

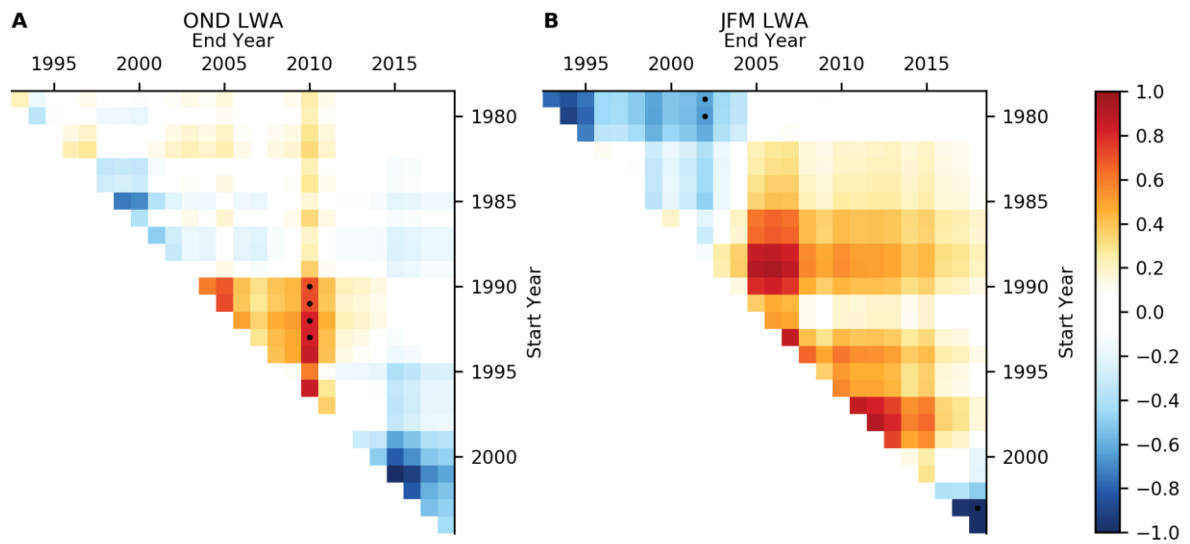

Fig. S2. Observed trends in waviness over the North American–Atlantic region. As in Fig. 2 (C,D) but averaged over the North America-Atlantic sector (220°E-360°E).

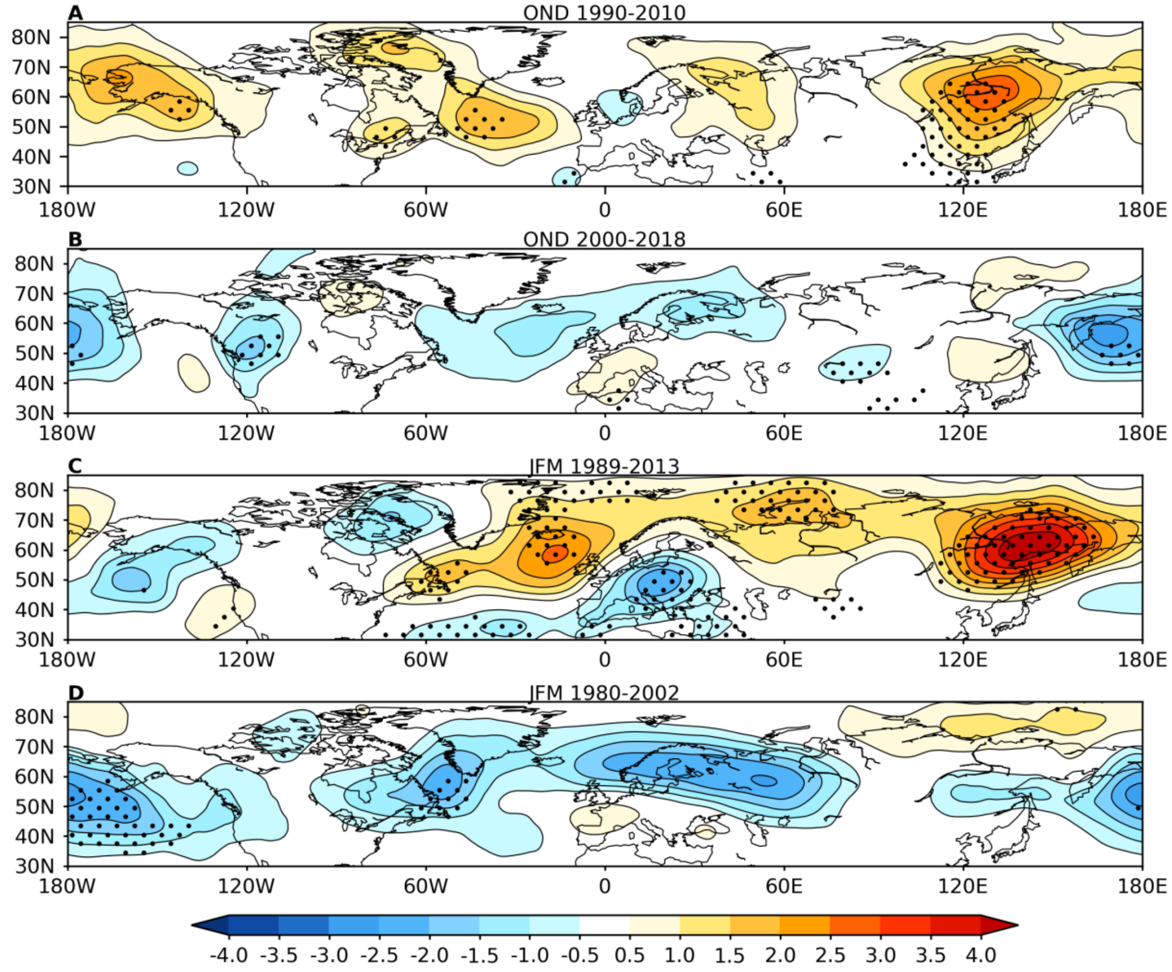

Fig. S3. Short-term observed trends in waviness. (**A**) Observed trends in LWA (10^7 m^2/decade) calculated over 1990-2010 during OND. Stippling indicates trends that a statistically significant at the 95% confidence level. (**B**) As in (A) but for 2000-2018. (**C**) As in (A) but for 1989-2013 during JFM. (**D**) As in (C), but for 1980-2002.

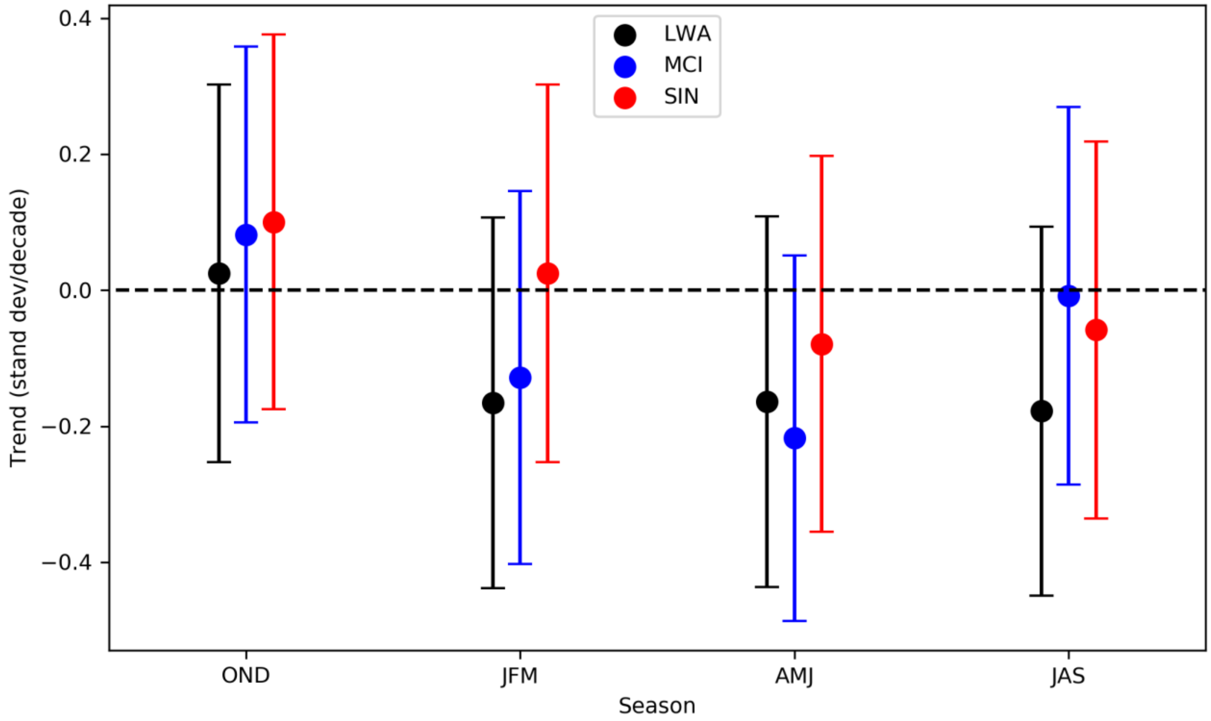

Fig. S4. Observed trends in waviness from additional metrics across all seasons. Observed trends in LWA (black), meridional circulation index (MCI; blue) and sinuosity (SIN; red) calculated from 1979-2018 for each season. All trends have units of standard deviations/decade. Error bars represent the 95% confidence interval.

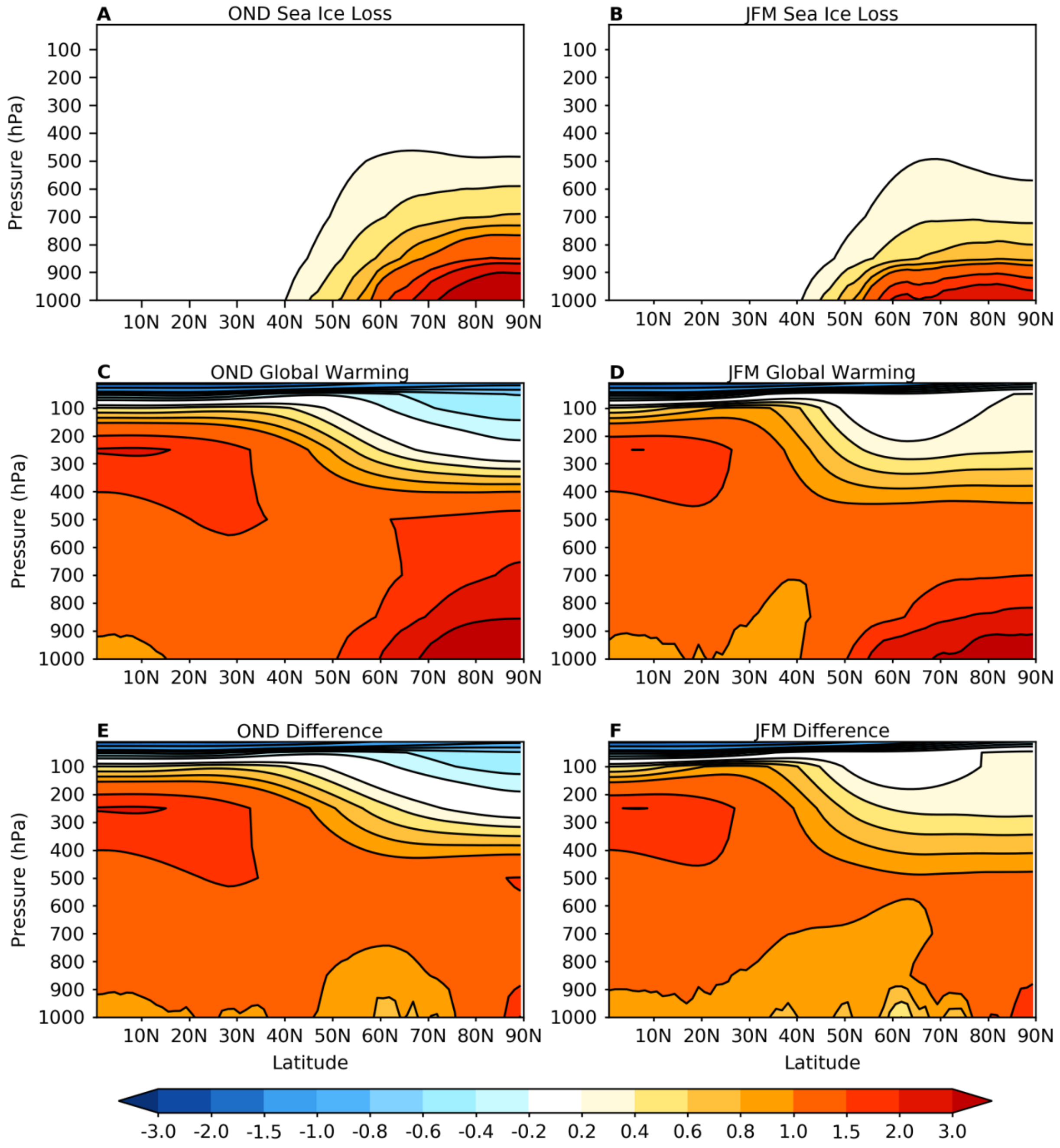

Fig. S5. Zonal mean temperature response to sea ice loss and global warming. (**A**) The zonal-mean air temperature response in the simulations with reduced sea ice in isolation for OND. (**C**) As in (A) but for simulations in response to global warming. (**E**) The difference between (C) and (A). (**B**,**D**,**F**) As in (A,C,D), but for JFM.

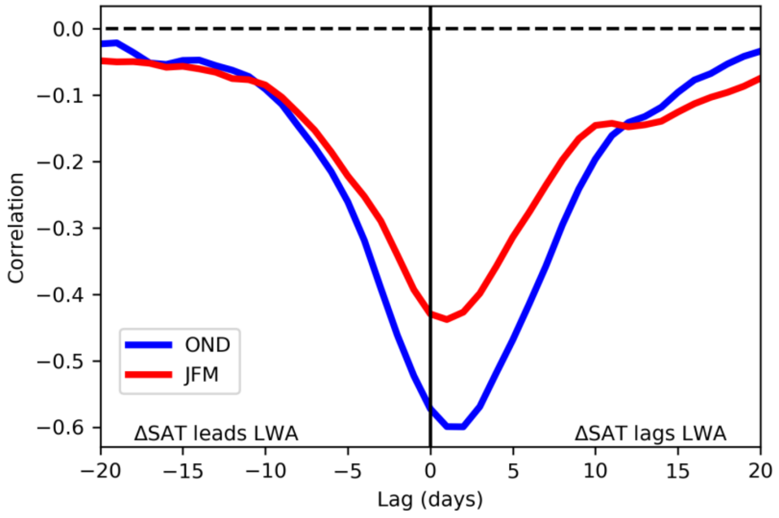

Fig. S6. Daily lead-lag correlations between waviness and meridional near-surface temperature gradient. Lead-lag correlations between LWA and ΔSAT from daily averaged data for OND (blue) and JFM (red) from the HadGEM2 model. Negative lag indicates the ΔSAT is leading the LWA and positive lag indicates the ΔSAT lags behind the LWA.
